# Development of a Tunable Dextran-PCL Biomaterial Photoink for High-Resolution DLP 3D Printing in Biomedical Applications

**DOI:** 10.1021/acsami.5c18856

**Published:** 2025-11-06

**Authors:** Inês C. P. Escobar, Leonor Chaves, Carlos T. B. Paula, Patrícia Pereira, Arménio C. Serra, Jorge F. J. Coelho

**Affiliations:** † CEMMPRE, ARISE, Department of Chemical Engineering, 56069 University of Coimbra, Rua Sílvio Lima, Polo II, Coimbra 3030-790, Portugal; ‡ IPN, Instituto Pedro Nunes, Associação Para a Inovação E Desenvolvimento Em Ciência E Tecnologia, Rua Pedro Nunes, Coimbra 3030-199, Portugal

**Keywords:** 3D printing, digital light processing (DLP), biomaterial photoink, dextran, poly(ε-caprolactone)

## Abstract

Digital light processing (DLP) is a technique that offers higher printing speeds and high spatial resolution compared to other additive manufacturing techniques. However, in biomedical applications, the biomaterials used do not provide a good balance between biocompatibility, mechanical performance, and controlled degradation. To overcome these limitations, this study aims to develop a biomaterial photoink formulation using two FDA-approved polymers. Dextran, which has high biocompatibility, was modified with glycidyl methacrylate to introduce photoreactive groups, and poly­(ε-caprolactone) (PCL), a biodegradable synthetic polymer known for its mechanical reinforcement properties and slower degradation rate, was functionalized with 2-isocyanatoethyl methacrylate. To optimize the formulation, various parameters were systematically investigated, including different polymer concentrations (10–40% w/v), concentrations of photoabsorber (0.075–0.2%) and photoinitiator (LAP) (0.3–1.25%), exposure time (13–21 s), and light intensity (45–65%). Once the optimal composition of the biomaterial photoink was determined, the effect of different polymer contents on the physicochemical, mechanical, and cytotoxic properties of the printed structures was investigated. It was found that increasing the proportion of PCL in the biomaterial photoink can lead to a slower degradation rate, reduced swelling capacity, and improved mechanical properties; however, cytocompatibility was negatively affected after 14 days of indirect contact. Direct cytotoxicity testing revealed cytocompatibility after 3 days. This study enabled the development of a highly tunable biomaterial photoink that can be adapted to different biomedical application requirements. The optimized biomaterial photoink exhibited good mechanical properties, lower viscosity, and excellent printability, enabling the printing of complex geometries (e.g., tubes), including hollow structures.

## Graphical abstract

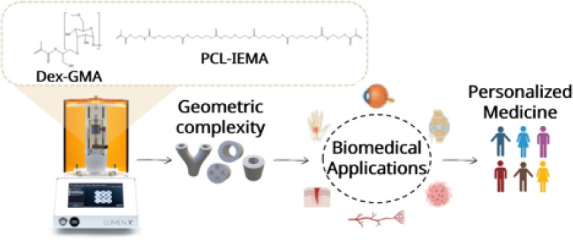

## 1 Introduction

Additive manufacturing (AM) has evolved into an advanced technology that can produce complex 3D geometries and structures directly from 3D models^1,2^ created using computer-aided design (CAD) software.^3^ These technologies have gained enormous importance for biomedical applications due to their high degree of customization and scalability. They enable the production of personalized medical devices and implants that are adapted to the patient’s individual anatomy and offer innovative approaches for drug delivery and tissue engineering.^1,2^

One of the most promising 3D printing processes is vat photopolymerization (VPP), in which a vat (or tank) is filled with liquid resin and cured with a light source. It offers significant advantages in terms of print resolution (down to the μm range), the creation of complex structures, and efficiency.^4–6^ Among vat photopolymerization technologies, digital light processing (DLP) has recently gained prominence due to its processing speed and high resolution.^4–7^ DLP is a light-based printing technology that uses a digital micromirror device (DMD) projector to cure and solidify an entire monomer layer at once, enabling better print resolution than other AM technologies.^5–10^ However, DLP has some limitations, such as the relatively high cost and limited availability of printing materials.^3^ Therefore, the development of new biomaterial photoinks is urgently needed.^11^

In recent years, various biopolymers have been used to develop acrylate- and methacrylate-based photoinks that are compatible with DLP.^12^ These include natural and synthetic polymers such as gelatin methacrylate (GelMA),^13,14^ polyethylene glycol dimethacrylate/-diacrylate (PEGDMA/PEGDA),^15,16^ silk fibroin methacrylate (SF-MA),^17–19^ and acrylated forms of hyaluronic acid (HAMA).^20^ However, the significant swelling and poor mechanical properties of these polymers still pose a challenge for 3D printing scaffolds with high accuracy.^12^

To overcome this problem, this study used a combination of natural and synthetic polymers, both approved by the Food and Drug Administration (FDA) and modified with double bonds to enable a rapid photocuring reaction. Dextran (Dex) was chosen because it is a biocompatible and biodegradable material that is already used in several biomedical applications.^21^ However, it also has problems such as poor mechanical properties, rapid degradation *in vivo*, and limited structural stability.^22^ To address these limitations, PCL has been incorporated into the formulation to control the degradation rate and improve the final mechanical properties.^23^ PCL is considered nontoxic and biocompatible and is therefore widely used for resorbable sutures, scaffolds for regenerative therapies, and drug delivery applications.^24^ Moreover, studies have shown that the combination of Dex and PCL can improve cell adhesion and provide mechanical support for vascularized bone tissue engineering^25^ and peripheral nerve regeneration.^21^

In addition to the polymers, a key element of the printing ink is the photoinitiator (PI), which plays a decisive role in photopolymerization during the printing process. When irradiated with light of a suitable wavelength, the PI generates free radicals that initiate the photopolymerization of the biomaterial photoink.^8^ Lithium phenyl (2,4,6-trimethylbenzoyl)­phosphinate (LAP) is often used as a PI for DLP printing due to its hydrophilic properties, low cytotoxicity, and fast initiation kinetics.^11^ In addition to the initiator, another essential element is the photoabsorber (PA).^26^ These molecules help prevent overcuring of layers beyond the focal plane by absorbing scattered light, which can lead to unwanted cross-linking of the material, thus improving the resolution of the 3D-printed structure in the XY plane.^27^ The most commonly used PAs are benzotriazole derivatives and general food dyes, such as tartrazine.^9^ Optimizing the ratio of PI and PA during DLP printing is therefore crucial for achieving successful results.^28^ Other parameters such as light output, printing time, and polymer ratio are also known to influence the dynamics of photopolymerization and are crucial for optimizing print resolution, mechanical properties, and physicochemical properties.^29^

To the best of our knowledge, this is the first report of a chemically modified Dex-PCL biomaterial photoink developed for DLP. This system demonstrates high printing fidelity and enables the tuning of degradation and mechanical properties by simply adjusting polymer ratios. This control is extremely important for tissue engineering, especially for bone scaffolds and peripheral nerve regeneration, where slower degradation and mechanical strength are required.

Accordingly, this work reports, for the first time, the successful optimization and characterization of a novel DLP-compatible biomaterial photoink using glycidyl methacrylate-modified dextran (Dex-GMA) and 2-isocyanatoethyl methacrylate-modified polycaprolactone (PCL-IEMA) as the main polymeric components.

The effects of different polymer ratios on the physicochemical and mechanical properties of the printed structures were investigated, as well as the effects of PI (LAP) and PA (tartrazine) on the printability. With the developed biomaterial photoink, a variety of complex structures with fine features, adjustable mechanical properties, and degradation rates could be printed. Finally, the cytotoxicity of the printed structures was demonstrated to ensure their suitability for future biomedical applications, drug delivery, and tissue engineering (Figure 1).

**Figure 1:**
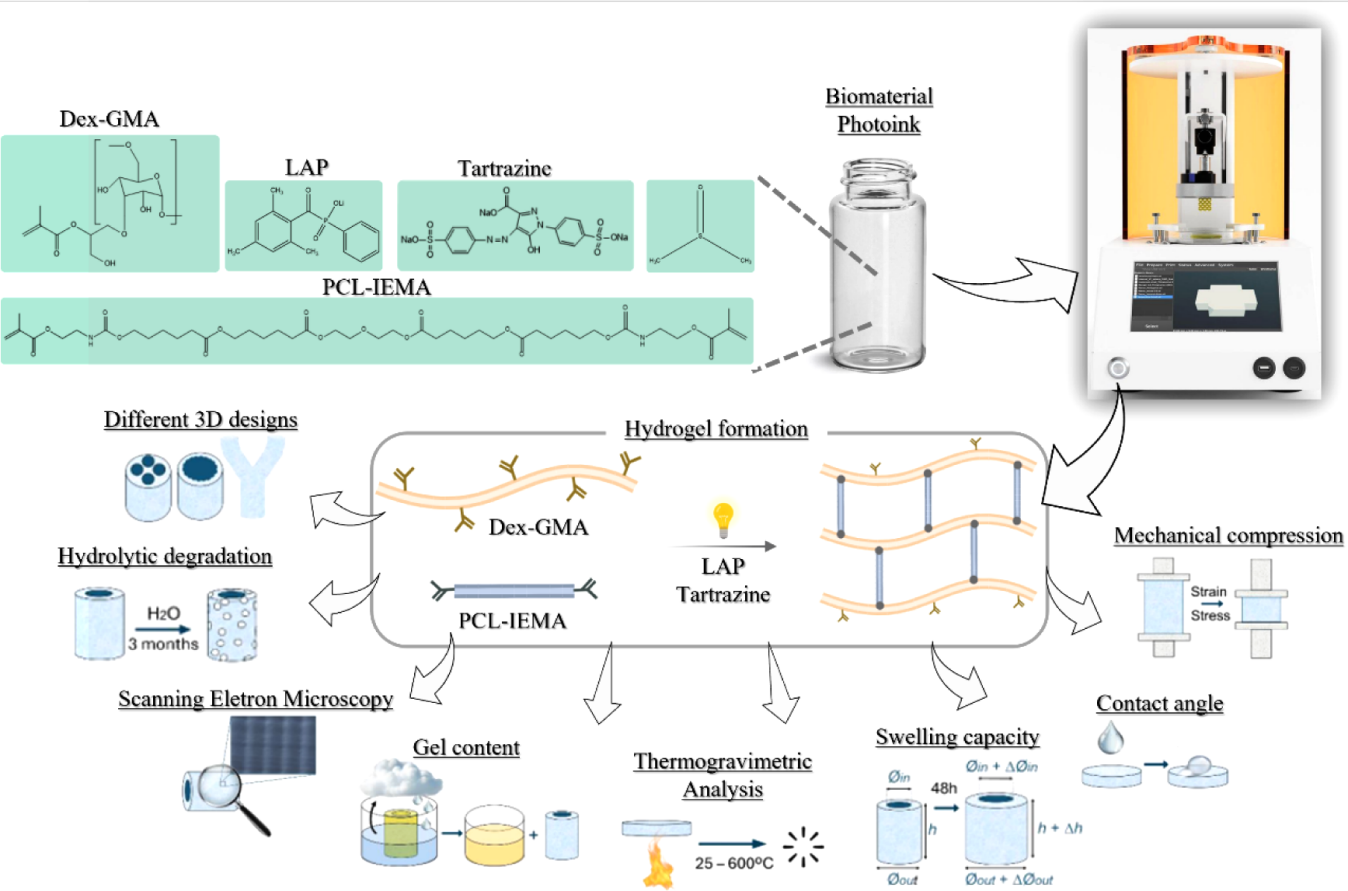
Schematic representation of the work: The biomaterial photoink formulation consists of Dex-GMA, PCL-IEMA, LAP, tartrazine, and DMSO. The printing parameters and composition of the biomaterial photoink were optimized to obtain high-resolution constructs with different 3D structures. The printed constructs were characterized in terms of their hydrolytic degradation (over 2 months in PBS), swelling ratio (after 48 h), gel content, morphology, and mechanical and thermal properties.

## 2 Experimental Section

### 2.1 Materials

Dextran (*M*_w_ ∼ 70 000 g·mol^–1^), poly­(ε-caprolactone)-diol (PCL-diol; *M*_w_ ∼ 530 g·mol^–1^), dibutyltin dilaurate 95% (C_32_H_64_O_4_Sn), dimethyl sulfoxide (DMSO) (*M*_w_ = 78.13 g·mol^–1^, C_2_H_6_OS), tetrahydrofuran (THF) (*M*_w_ = 72.11 g·mol^–1^, C_4_H_8_O), *n*-hexane (*M*_w_ = 86.18 g·mol^–1^,CH_3_(CH_2_)_4_CH_3_) and the *In Vitro* Toxicology Assay Kit, Resazurin-based were acquired from Sigma-Aldrich (St. Louis, Missouri, USA). Glycidyl methacrylate (GMA) 97% stabilized with 100 ppm 4-methoxyphenol (C_7_H_10_O_3_) was purchased from Thermo Scientific (Kandel, Belgium). 4-Dimethylaminopyridine (DMAP) (*M*_w_ = 122.17 g·mol^–1^, C_7_H_10_N_2_), 2-isocyanatoethyl methacrylate (IEMA) stabilized with BHT (*M*_w_ = 155.15 g·mol^–1^, C_7_H_9_NO_3_), lithium phenyl (2,4,6-trimethylbenzoyl)­phosphinate (LAP) (*M*_w_ = 294.10 g·mol^–1^, C_16_H_16_LiO_3_P), and Acid Yellow 23 (Tartrazine) (*M*_w_ = 534.36 g·mol^–1^, C_16_H_9_N_4_Na_3_O_9_S_2_) were obtained from TCI Europe (Zwijndrecht, Belgium). Deuterium oxide (D_2_O) and DMSO-D_6_ were acquired from EurisoTop (Saint-Aubin, France). Sodium azide was purchased from Panreac (Barcelona, Spain). Dialysis membranes (Spectra/Por) were purchased from Thermo Fisher Scientific. All of the reagents were used as received.

### 2.2 Preparation and Characterization of Dex-GMA and PCL-IEMA

#### 2.2.1 Functionalization of Dextran with GMA (Dex-GMA)

The functionalization of dextran (Dex) with GMA was adapted from Pinho et al.^21^ In a round-bottom flask, 10 g (0.14 mmol) of Dex was dissolved in 90 mL of DMSO and left overnight in a bath at 30 °C until complete dissolution. Then, 2 g (16.4 mmol) of DMAP and 8.20 mL (61.7 mmol) of GMA were added to the reaction mixture and allowed to react for 8 h under a nitrogen atmosphere. The mixture was then neutralized with a 37% (w/w) HCl solution and subsequently dialyzed against distilled water for at least 2 days. The reaction product (Dex-GMA) was obtained by lyophilization.

#### 2.2.2 Functionalization of PCL-Diol with IEMA (PCL-IEMA)

The functionalization of PCL-diol with IEMA was adapted from Pinho et al.^21^ First, 8.8 g (16.6 mmol) PCL-diol was dissolved in 120 mL THF in a round-bottom flask. The solution was kept in a bath at 40 °C under a nitrogen atmosphere. After complete dissolution of PCL, 4.62 mL (32.7 mmol) of IEMA and 3 drops of dibutyltin dilaurate were added, and the reaction was allowed to proceed for 24 h. After this time, PCL-IEMA was obtained by precipitation in *n*-hexane. The product was dried at room temperature in a fume hood to remove any residual solvent.

#### 2.2.3 Proton Nuclear Magnetic Resonance (^1^H NMR) Spectroscopy

The polymeric precursors Dex-GMA and PCL-diol were characterized by ^1^H-NMR spectroscopy. The ^1^H-NMR spectra were recorded at 25 °C by using a Bruker Avance III 400 MHz spectrometer coupled to a 5 mm triple detection TIX probe. For Dex-GMA, the sample was dissolved in D_2_O and a specific pulse angle of 87.7° with a relaxation delay of 30 s was used. The water signal at 4.8 ppm was eliminated by solvent suppression with decoupling.^21^ The coupling power was adjusted to a value at which the intensity of the anomeric proton signal was not affected. DMSO-*d*_6_ was used as the solvent for PCL-IEMA.

### 2.3 Preparation of Biomaterial Photoink and 3D Structures

#### 2.3.1 Preparation of Biomaterial Photoink

Dex-GMA (0.25, 0.5, and 0.75 g) and PCL-IEMA (0.25, 0.5, and 0.75 g) were dissolved in DMSO (5 mL) to obtain a total polymer concentration of 20% (w/v), corresponding to Dex:PCL mass ratios of 25:75 (25Dex:75PCL), 50:50 (50Dex:50PCL), and 75:25 (75Dex:25PCL), respectively. Then, the photoinitiator LAP and the photoabsorber tartrazine were added to each formulation at final concentrations of 0.4% (w/v) and 0.1% (w/v), respectively.

#### 2.3.2 Fabrication of 3D-Printed Constructs by DLP

The polymer precursors Dex-GMA and PCL-diol were 3D-printed using a LUMEN X^+^ system from CELLINK (Gothenburg, Sweden). The 3D CAD model was designed using Autodesk Fusion 360 software, converted to an STL file, and sliced into 100 μm layers. The printing parameters were a projector power of 55% (equivalent to 21 mW/cm^2^), an exposure time between 19 and 20 s, and a base exposure time factor (BEF) of 3× at a wavelength of 405 nm. After printing, the printed constructs were placed on absorbent paper for 1 day to remove excess DMSO and allow for a milder shrinking process. The 3D-printed constructs were then immersed in distilled water for 3 days to remove the yellow Tartrazine dye and residual DMSO. The DLP-printed constructs were labeled as 25Dex:75PCL, 50Dex:50PCL, and 75Dex:25PCL if they contained 25% Dex-GMA and 75% PCL-IEMA, 50% Dex-GMA and 50% PCL-IEMA, and 75% Dex-GMA and 25% PCL-IEMA, respectively.

#### 2.3.3 Optimization of the Formulation

In order to optimize the printing conditions, the influence of various parameters such as exposure time (s), light intensity (%), and concentration of LAP and tartrazine on the resolution of the printed structures were first investigated. The formulation 25Dex:75PCL was used, and cubes (edge length (ED): 5 mm × 5 mm × 5 mm) were printed under specific conditions and classified using a three-grade colormap.

After optimizing the printing parameters, three different polymer concentrations, 10, 20, and 40% (w/v), were tested to determine the most suitable concentration for the printed constructs in terms of dimensional stability, ink viscosity, opacity, and final quality after washing. A 25Dex:75PCL formulation was printed in the shape of a cube (ED: 5 mm × 5 mm × 5 mm), dried on absorbent paper for 1 day, and washed in distilled water for 3 days.

#### 2.3.4 Evaluation of the Printability of the 3D Structures

The 25Dex:75PCL, 50Dex:50PCL, and 75Dex:25PCL formulations were printed with different exposure times: 19.25, 19.5, and 19.75 s, respectively. A 3D CAD model of a tube was created (height (*h*) × Ø_in_ × Ø_out_: 5 mm × 2.5 mm × 5 mm). The printability ratio was calculated using eq 1:

1 $$Printability\;ratio\left(\%\right)=\frac{V_{p}}{V_{m}}\times 100$$

where *V*_p_ represents the final volume of the printed sample and *V*_m_ represents the volume of the CAD model.^11^ Measurements were made for each biomaterial photoink formulation in triplicate.

#### 2.3.5 Shrinkage of the 3D-Printed Structure Assessment

After 3D printing with formulations 25Dex:75PCL, 50Dex:50PCL, and 75Dex:25PCL, the printed tubes were dried on absorbent paper for 1 day, then soaked in distilled water for 3 days, and finally dried in a vacuum oven at 50 °C until a constant weight was achieved. The associated shrinkage of the printed constructs is estimated using eq 2:

2 $$Shrinkage\left(\%\right)=\frac{D_{p}-D_{shk}}{D_{p}}\times 100$$

where *D*_p_ represents the final dimension of the printed sample and *D*_shk_ is related to the dimension after drying.^30^ Measurements were taken for each sample subjected to the printability test, and shrinkage was calculated for different drying and washing stages.

#### 2.3.6 Gel Content

The gel content of the printed tubes was determined by a Soxhlet extraction. It consisted of two cycles of 24 h each; the first cycle was performed with THF, and the second cycle with distilled water to remove the excess non-cross-linked PCL-IEMA and Dex-GMA, respectively. The gel content was determined according to eq 3:

$$Gel\;content\left(\%\right)=\frac{W_{s}}{W_{0}}\times 100$$ 3

where *W*_0_ is the initial weight of the dry structure after printing and *W*_s_ represents the final weight after Soxhlet extraction.^21^

#### 2.3.7 Thermal Analysis

The thermal properties of the printed cylinders (*h* × Ø_out_: 2 mm × 5 mm) were analyzed by thermogravimetric analysis (TGA). Thermal stability studies were performed on a NETZSCH STA 449 F5 (Netzsch, Germany) instrument, employing a heating rate of 10 °C·min^– 1^ over a temperature range of 25–600 °C under a nitrogen purge.

#### 2.3.8 Compression Tests

Compression tests were performed on a Hegewald & Peschke Inspekt Solo 500N Universal Testing Machine (LabMaster software) for printed cylinders (*h* × Ø_out_: 9 mm × 6 mm). The samples were swollen in distilled water to their maximum capacity and compressed at room temperature at a constant rate of 2 mm/min with a maximum percentage strain of 80%. The compression modulus (Young’s modulus) was calculated based on the slope of the linear range from 2 to 10% strain. All compression tests were repeated five times for each formulation, and the data were averaged to determine the final compressive stress–strain values.

#### 2.3.9 Swelling Capacity

The dried printed tubes (*h* × Ø_in_ × Ø_out_: 5 mm × 2.5 mm × 5 mm) were immersed in 1 mL of PBS solution (pH 7.4) at 37 °C for 48 h. At predetermined times, the swollen samples were removed from the solution and dried with absorbent paper, and their weight was recorded. The procedure was repeated until an equilibrium swelling capacity was reached. The swelling capacity of the printed constructs was calculated using eq 4:

4 $$Swelling\;capacity\left(\%\right)=\left(\frac{W_{s}-W_{d}}{W_{d}}\right)\times 100$$

where *W*_d_ is the initial weight of the dried samples before immersion in PBS and *W*_s_ is the final weight of the swollen samples. Six replicates were conducted for each biomaterial photoink formulation.

#### 2.3.10 *In Vitro* Hydrolytic Degradation

*In vitro* hydrolytic degradation tests of the printed tubes were performed in PBS solution (pH 7.4) with 2 wt % sodium azide at 37 °C for 2 months.^21^ At predetermined intervals, the printed constructs were removed from the PBS solution and rinsed three times with distilled water. Then, the samples were dried for 2 days under vacuum at 50 °C until their weight stabilized. The degree of degradation was calculated according to eq 5:

5 $$Weight\;loss\left(\%\right)=\left(W_{0}-W_{t}\right)/W_{0}\times 100$$

where *W*_0_ is the initial weight of the dry sample before immersion and *W*_t_ is the final weight of the sample after immersion in PBS and subsequent drying. Six replicates were conducted for each photoink formulation and each day of analysis.

#### 2.3.11 Scanning Electron Microscopy

SEM analysis was performed using scanning electron microscopy (SEM) on ZEISS MERLIN Compact/VPCompact, Gemini II (FESEM, ZEISS MERLIN, Oberkochen, Germany) to assess layer definition, printed integrity, presence of artifacts, and the impact of degradation on the integrity of the printed constructs. The samples were oven-dried at 50 °C under vacuum and then coated with gold before analysis.

#### 2.3.12 Contact Angle

Contact angle measurements were performed using a OneAttension contact angle goniometer (Biolin Scientific, Finland). Distilled water (3 μL) was placed on the printed cylinders (*h* × Ø_out_: 1.5 mm × 20 mm). Contact angles were measured for three replicates on three different formulation surfaces using OneAttension software.

#### 2.3.13 Cytotoxicity Tests: Indirect Test

The cytotoxicity was determined using an indirect method based on ISO 10993-5 to analyze the degradation products of the printed structures. For this purpose, disk-shaped 3D CAD models (*h* × Ø_out_: 1.5 mm × 10 mm) were printed and placed on absorbent paper for 1 day and then washed in distilled water for 3 days. After washing, the samples were sterilized with UV light for 30 min before *in vitro* testing. The sterilized printed structures were immersed in a culture medium and stored at 37 °C for 1, 2, 3, 7, and 14 days. Human embryonic kidney cells (HEK293T) and mouse neuroblasts (Neuro-2a) were seeded in 96-well cell culture plates in 100 μL of the DMEM High Glucose Medium at a density of 1 × 10^4^ cells/well. After 24 h of culture in a humidified atmosphere with 5% CO_2_ at 37 °C, the medium was removed, replaced with 100 μL of the extracts from the printed structures, and incubated for 24 h. Cells cultured in a normal medium served as negative controls for 100% viability, and cells cultured in 10% DMSO served as positive controls for 100% cytotoxicity. The cytotoxicity of the degradation products was determined by quantitative analysis using an *In Vitro* Toxicology Assay Kit, Resazurin-based (Sigma-Aldrich). After 24 h, cells were incubated for 4 h with 10% Resazurin in the culture medium, and the absorbance was measured at 570 and 600 nm using a microplate reader. Cell viability was calculated as the percentage of survival compared to untreated cells, which were assumed to have 100% viability.

#### 2.3.14 Cytotoxicity Tests: Direct Test

To evaluate the direct contact of the materials with the cells, HEK293T cell cultures, at a density of 2.5 × 10^4^ cells/well, were seeded on top of hydrogel disk samples. The cell-seeded hydrogels were maintained at 37 °C and 5% CO_2_ for 3 days. On the test day (day 3), the culture medium was removed and replaced with a mixture of the culture medium and a Resazurin-based *In Vitro* Toxicology Assay Kit (Sigma-Aldrich). Briefly, 500 μL of the serum-free culture medium containing Resazurin reagent (10%) was added to each well and incubated for 4 h at 37 °C in a 5% CO_2_ atmosphere. Then, 100 μL from each well (in quadruplicate) was transferred to a 96-well plate, and the absorbance was measured at 570 nm (normalized to a value of 600 nm) by using a BioTek Synergy HTX Multimode Reader (Agilent). A negative control (untreated cells) was included, i.e., cells cultured without exposure to the samples. All samples were tested in triplicate. Cell viability was calculated as the percentage of viable cells relative to untreated cells, which were considered to be 100% viable.

#### 2.3.15 Statistical Analysis

All data were obtained from at least three parallel samples and are expressed as mean ± SD. Significant differences between experimental groups were determined by two-way analysis of variance (Tukey’s multiple comparison test) using GraphPad Prism 10.4.0 software (GraphPad Software Inc., La Jolla, CA). A *p*-value lower than 0.05 (**** *p* < 0.0001) was considered statistically significant.

## 3 Results and Discussion

### 3.1 Characterization of Dex-GMA and PCL-IEMA Polymeric Precursors

In the development of a biomaterial photoink, polymerizable components with methacrylate and acrylate groups are incorporated into polymer structures to enable rapid and selective solidification and to form a cross-linked matrix. For this purpose, Dex and PCL-diol were modified with GMA and IEMA, respectively, to incorporate methacrylate groups. The functionalization and the degree of substitution of the Dex-GMA and PCL-IEMA samples were determined by ^1^H NMR spectroscopy (Figure S1).

The Dex-GMA spectrum (Figure S1b) shows the presence of methyl protons belonging to the methacrylate group of GMA at δ 1.9 ppm, the Dex backbone peaks from δ 3.3 ppm to δ 4.0 ppm, the protons of the double bond at δ 5.7 ppm and δ 6.1 ppm, and contributions of the anomeric proton of Dex between δ 4.87 and 5.13 ppm, confirming the successful synthesis of Dex-GMA.^23^ The degree of substitution of dextran was between 0.88 and 1.07 mol of GMA per dextran repetition unit, calculated by integrating the double bond protons of the GMA and the anomeric proton of the dextran using the equation shown in equation S1.

In the case of PCL-diol, functionalization was carried out with IEMA. PCL-IEMA was synthesized by a reaction between the terminal hydroxyl groups of PCL and the isocyanate groups of IEMA to form urethane bonds. The ^1^H NMR spectrum of PCL-IEMA (Figure S1d) shows the presence of the −NH group of the urethane bond at δ 7.2 ppm; the protons of the double bond are assigned at δ 5.7–6.1 ppm; the −CH_3_ protons of the terminal groups of IEMA linked to PCL appear at δ 1.9 ppm, as well as the protons in the backbone chain of PCL (δ 1.3, δ 1.6 ppm, δ 2.3 ppm, and δ 4.0 ppm). These results confirm the synthesis of PCL-IEMA.^23,31^ The degree of functionalization of PCL-diol was between 85 and 97%, calculated by considering the olefinic protons of IEMA and the −CH_2_ protons of the repeating unit in the PCL backbone (equation S2).

### 3.2 Preparation of Biomaterial Photoink

#### 3.2.1 Optimization of the Formulation

The concentration of the photoinitiator and photoabsorber influences the printability of 3D-printed structures in terms of the quality of the structure, resolution, sample size, timing, and degree of toxicity.^32^ In addition, for cell-laden prints, exposure times and light intensities can also affect the viability of cells during the printing process.^32–34^

A series of tests were developed consisting of different experiments to evaluate the influence of light intensity, exposure time, and concentrations of photoinitiator and photoabsorber. Several experiments were carried out to determine which experiment gave the best results, and these are summarized in Table 1.

**Table 1:** Summary of the Optimization Trials Using 20% (w/v) of Polymer Concentration and Layer Height = 100 μm: Respective Parameters Varied and Fixed in Each Trial and Relevant Observations

Trial	Polymers ratio	Irradiance (mW/cm^2^)	LAP concentration (% (w/v))	Tartrazine concentration (% (w/v))	Exposure time (s)	Observations
1	25Dex:75PCL	21	0.625	0.075–0.2	13–21	Best results with 0.1–0.15% tartrazine (19–21 s)
2	25Dex:75PCL	21	0.3–1.25	0.075–0.15	13–21	Best printability results with 0.4% LAP + 0.1% tartrazine (17–21 s)
3	25Dex:75PCL	16.5–24.1	0.4	0.1	15–21	Most accepted value: 21 mW/cm^2^, 17–21 s
4	100Dex and 100PCL	21	0.4	0.1	17 and 23	Single polymer unsuitable for printing

Using a simple three-level color map (Figure 2), the printed structures were classified into poor resolution (red: printed layers overlap, strong scattering effects, and impaired cube dimensions), medium resolution (yellow: printed layers overlap and/or less scattering effects), and good resolution (green: printed layers very well aligned to the naked eye with only minimal scattering). The three-level color map was constructed primarily through visual inspection, focusing on the resolution of the printed cubes rather than just their dimensions.

**Figure 2:**
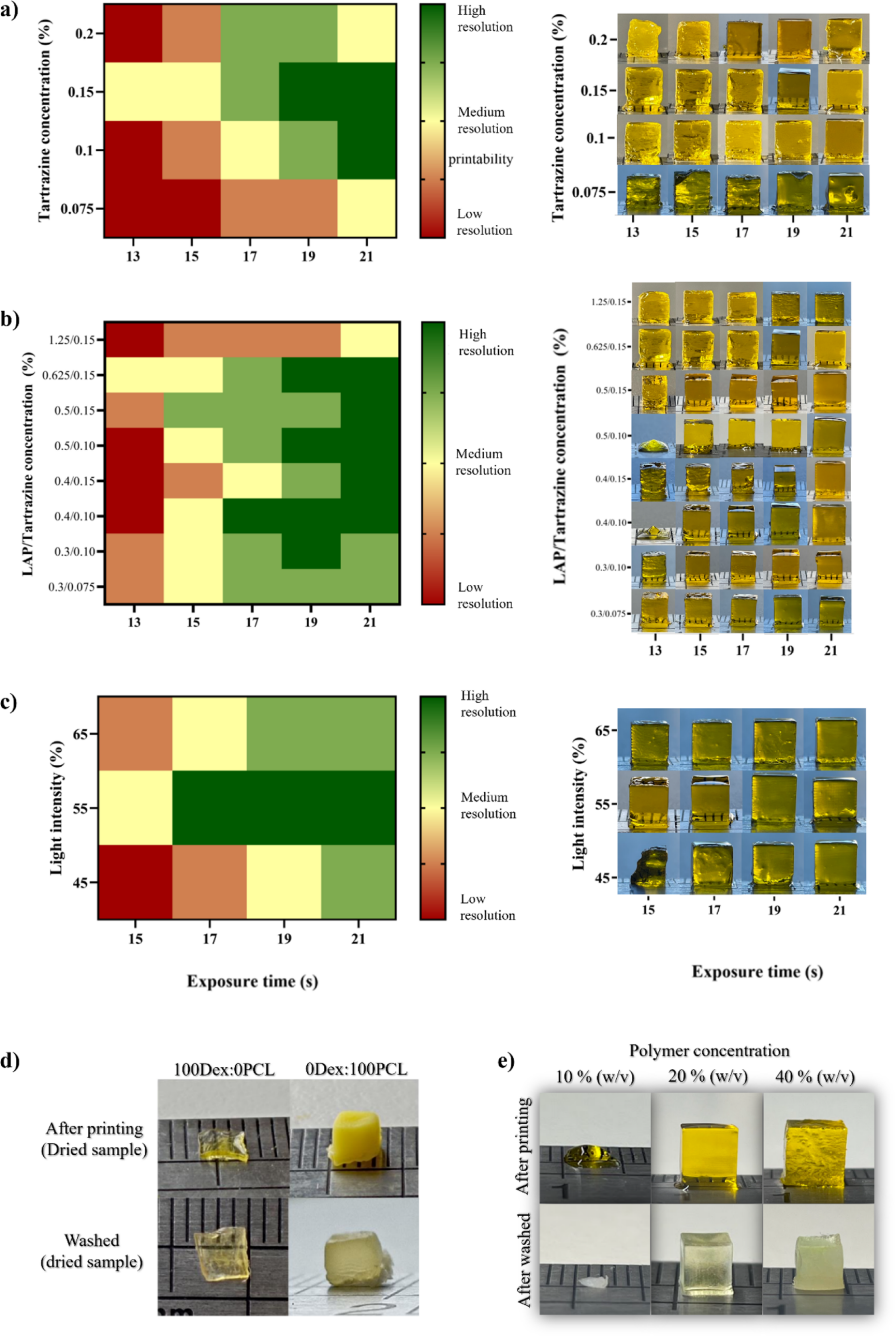
Optimization of the formulation: A three-grade colormap of 3D-printed cubes (ED: 5 mm × 5 mm × 5 mm) with a 25Dex:75PCL formulation and respective images of printed structures, using 20% (w/v) of polymer concentration, exposure times of 13 to 21 s, irradiance 21 mW/cm^2^ and a layer height of 100 μm varying: (a) the concentration of tartrazine (Trial 1: 0.625% (w/v) LAP, 0.075–0.2% (w/v) tartrazine); (b) concentration of LAP (Trial 2:0.3–1.25% (w/v) LAP, 0.075–0.15% (w/v) tartrazine); (c) light intensities (Trial 3: 0.4% (w/v) LAP, 0.1% (w/v) tartrazine, irradiance 16.5–24.1 mW/cm^2^); (d) polymer composition (Trial 4) for 100Dex:0PCL and 0Dex:100PCL, with exposure times of 17 and 23 s, respectively (20% (w/v), of polymer concentration, 0.4% (w/v) LAP, 0.1% (w/v) tartrazine); and (e) polymer concentrations (formulation 25Dex:75PCL, 10%–40% (w/v) of polymer concentration, 0.4% (w/v) LAP, 0.1% (*w/v*) tartrazine).

The role of tartrazine concentration for the same LAP concentration (Trial 1, Figure 2a) was evaluated at different exposure times. It was found that concentrations of 0.625% LAP and 0.15% tartrazine at 19–21s and 0.625% LAP and 0.1% tartrazine at 21 s could produce the most satisfactory cubes with good resolution. A slight increase in the amount of tartrazine to 0.2%, medium to low resolution was observed in all printed structures. With a lower amount of tartrazine (0.075%), the results were unsatisfactory, as most of the structures showed low resolution, and only with higher exposure time (21 s) did the printed structures achieve medium resolution.

The effect of varying the LAP concentration was then studied (Trial 2, Figure 2b). The LAP concentration was changed, and the photoabsorber concentration was also adjusted. The results showed that for some of the LAP concentrations, high printability occurs, especially over 17 s. The worst printabilities were obtained for 1.25% LAP and 0.15% tartrazine. Nevertheless, LAP concentrations are usually kept between 0.1 and 0.6%, to also minimize their cytotoxicity.^35^

The three-grade colormap shows that the resolution of printability increases with decreasing amounts of LAP, with better results for 0.4% LAP and 0.1% tartrazine (17 to 21 s).

The effect of light intensity (Trial 3, Figure 2c) was also investigated. The light intensity of 55% corresponding to an irradiance of 21 mW/cm^2^ during 17–21 s is the most accepted value, and with 15 s of exposure time, only a medium resolution is achieved. The light intensity of 45% (irradiance 16.5 mW/cm^2^ during 15–19 s did not show high printability but only good printability for 21 s. For 65% light intensity (irradiance 24.1 mW/cm^2^), the structures could only be printed after 19 s per layer but without perfect resolution. The light intensity with the widest range of exposure times for prints with medium to high resolution is therefore 55%. Compared to studies reported in the literature that use the same wavelength and typically report light intensities between 35 mW/cm^2^ to 60 mW/cm^2^ and exposure times of 10–15 s, our study shows the advantage that high-quality prints can be achieved with lower light intensity.^36–38^

The influence of polymer composition (Trial 4, Figure 2d) was evaluated to understand the role of each polymer on printability and structural definition. Two inks, consisting of only one type of polymer—100Dex:0PCL and 0Dex:100PCL—were printed with an exposure time of 17 and 23 s per layer, respectively. The 100Dex:0PCL formulation was unable to form a structure beyond the first few layers at both exposure times, indicating that Dex alone does not provide sufficient mechanical stability, while 0Dex:100PCL printed a malleable cube shape (at 23 s) with extreme shrinkage immediately after printing. These studies show that PCL provides mechanical support to the structure, and Dex contributes to structural definition. The combination of these elements is essential for obtaining high-resolution structures.

When developing a functional biomaterial photoink, the polymer concentration is a crucial parameter that must be investigated. This parameter affects the viscosity of the ink and can consequently influence the polymerization process and the dimensional stability of the final structure. After optimizing the printing conditions, different polymer concentrations were tested to determine which one was best suited for use. In Figure 2e, the concentration of 10% (w/v) is so low that it leads to a lower cross-linking degree and thus poorer printability.^39^ At 40% (w/v), the hydrogel becomes very compact and dense, making it difficult for solvents to leave the hydrogel network and resulting in an opaque structure. The polymer concentration of 20% (w/v) proved to be the best option, offering optimal printability and transparency. Moreover, the solution presented a viscosity suitable for DLP technology and produced structures with good print resolution.^40^ For this reason, a polymer concentration of 20% (w/v) was used for printing in all further experiments.

Although no study has reported the use of PCL-IEMA and Dex-GMA for DLP printing, the polymer concentration determined is in line with the concentrations reported in the literature for other biopolymers such as GelMA^13,15^ and SF-MA (between 5 and 25%).^17,19^

Under the conditions tested, a combination of 20% polymer concentration, 0.4% LAP, and 0.1% tartrazine, together with 55% light intensity (21 mW/cm^2^) and an exposure time of 17–21 s per layer, resulted in the best print fidelity.

#### 3.2.2 Printability Ratio and Structure Shrinkage

The printability ratio represents the difference between the planned CAD model and the final printed structure. The closer the printability ratio is to 100%, the greater the match with the original design.^11^ After optimizing the printing conditions for the PI and PA concentrations, different ratios of PCL-IEMA and Dex-GMA were tested by adjusting the exposure time for the fabrication of hollow tubes, as these can be considered a complex structure that allows printability evaluation in the XY-axis (Figure 3a). The results are shown in Table 2.

**Table 2:** Printability Ratio for Different Formulations^a^ ^b^

	Dimensions after printing (mm)				
Formulation	Ø_out_	Ø_in_	*h*	*V*_p_	Printability ratio
CAD Digital Mask	5.00	2.50	5.00	73.63	-
25Dex:75PCL	4.95 ± 0.01	2.45 ± 0.04	4.96 ± 0.02	72.69 ± 0.10	98.72 ± 0.13
50Dex:50PCL	4.99 ± 0.01	2.50 ± 0.05	4.97 ± 0.04	72.85 ± 0.53	98.94 ± 0.72
75Dex:25PCL	4.99 ± 0.01	2.49 ± 0.04	4.99 ± 0.04	73.38 ± 0.21	99.66 ± 0.28

a Tubes (*h* × Ø_in_ × Ø_out_: 5 mm × 2.5 mm × 5 mm) were printed for all formulations under the following conditions : 20% (w/v) of polymer concentration, 0.4% (w/v) LAP, 0.1% (w/v) tartrazine, exposure time 19.25–19.75 s, irradiance 21 mW/cm², and layer height of 100 μm. Tube dimensions were measured using ImageJ software, and the printed volume (*V*_p_) was calculated and compared to the CAD model volume .

b Ø_out_: outer diameter of the printed tube, Ø_in_: inner diameter of the printed tube, *h*: height of the printed tube, and *V*_p_: volume of the printed tube.

**Figure 3:**
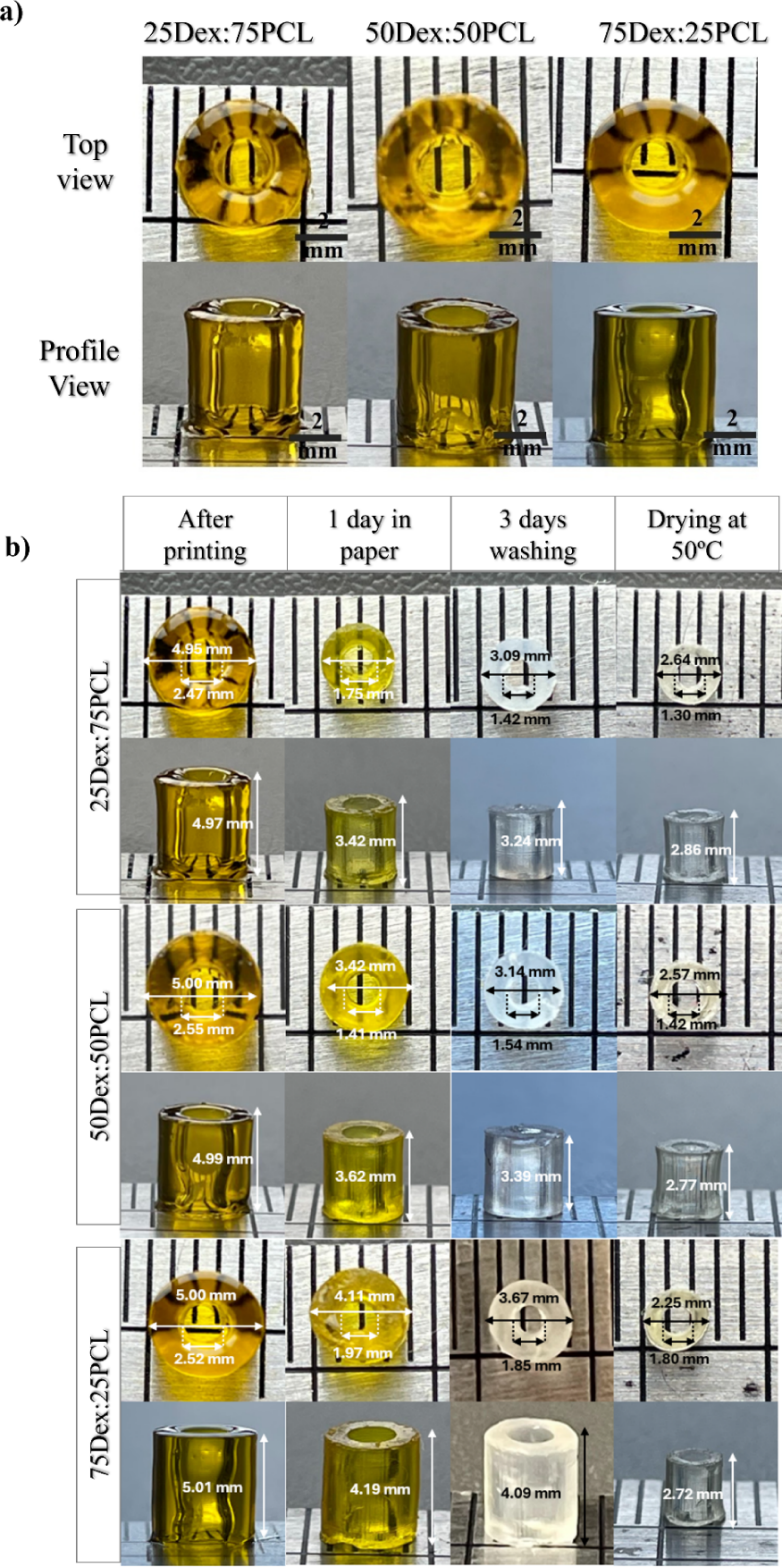
Printability and shrinkage of different formulations: (a) Image of all printed tubes following the printing process (25Dex:75PCL, 50Dex:50PCL, and 75Dex:25PCL with 19.25, 19.50, and 19.75 s of exposure time, respectively, with 0.4% (w/v) LAP, 0.1% (w/v) tartrazine, 21 mW/cm^2^ of light intensity, and a layer height of 100 μm); (b) shrinkage of each formulation during different drying and washing stages. Tube dimensions were measured by using ImageJ software.

The exposure time was adjusted for each formulation, and it was found that the presence of a higher amount of PCL-IEMA could lead to shorter exposure times. In Figure 3a, the top view and profile view of the printed tubes all exhibit excellent printability.

Despite the excellent printability, a shrinkage effect was observed in the printed tubes after successive printing tests. A first, more pronounced shrinkage stage is observed on the first day after drying on absorbent paper, followed by a second shrinkage stage after washing in distilled water, and the last shrinkage step during the drying process in the oven at 50 °C. The shrinkage values for each stage (drying on paper, washing, and drying at 50 °C) are shown in Table 3 and Figure 3b.

**Table 3:** Shrinkage Values of Printed Tube Dimensions for All Three Formulations at Each Washing/Drying Stage, Measured Relative to Postprinting Dimensions.^a^ ^b^

	Shrinkage (%) (1 day in paper)			Shrinkage (%) (3 days wash)			Shrinkage (%) (drying at 50 °C)		
Formulation	Ø_out_	Ø_in_	*h*	Ø_out_	Ø_in_	*h*	Ø_out_	Ø_in_	*h*
25Dex:75PCL	35.99 ± 2.15	33.48 ± 4.39	32.17 ± 2.18	42.23 ± 2.84	40.03 ± 1.84	36.22 ± 1.81	47.16 ± 0.20	45.51 ± 3.30	42.87 ± 0.39
50Dex:50PCL	31.04 ± 1.85	35.50 ± 6.88	26.49 ± 0.70	35.85 ± 0.98	38.19 ± 1.30	32.26 ± 0.39	48.93 ± 0.33	43.96 ± 1.42	43.65 ± 1.12
75Dex:25PCL	20.98 ± 2.43	20.06 ± 1.77	16.14 ± 1.08	25.92 ± 0.53	29.21 ± 2.36	20.35 ± 1.63	51.17 ± 2.73	32.03 ± 2.38	46.36 ± 0.38

a Tube dimensions were measured using ImageJ software.

b Ø_out_: outer diameter of the printed tube, Ø_in_: inner diameter of the printed tube, and *h*: height of the printed tube.

Table 3 shows that the greatest shrinkage occurs in the first drying phase, during which a large amount of solvent is transferred from the tube to the absorbent paper. The formulation with a higher PCL content showed a higher shrinkage percentage. It has been reported that acrylate- and methacrylate-based photoinks exhibit significant volume shrinkage after photopolymerization.^41^ This shrinkage, often exceeding 20%, typically occurs immediately after the photopolymerization process due to molecular rearrangement, which can result in weak interlayer bonding and distortion of the printed structure.^30,42^

During the final drying stage, the remaining solvent evaporates, and this trend is no longer observed, with shrinkage reaching about 45% for all dimensions and formulations.

In this study, high structural accuracy was observed immediately after printing (printability ratio close to 100%), with significant shrinkage occurring only after 1 day of drying on absorbent paper. However, based on these results, it is possible to adjust the dimensions of the CAD model for each formulation to achieve the desired final dimensions after the washing and drying process.

The higher shrinkage observed in formulations with increased PCL-IEMA content, compared with those with higher Dex-GMA content, can be attributed to the distinct molecular characteristics of the two components. PCL-IEMA, due to its relatively low molecular weight, increases the density of methacrylate groups in formulations, promoting a higher shrinkage value. In contrast, a large amount of Dex-GMA provides fewer methacrylate groups per unit mass, resulting in lower shrinkage values.^43^

#### 3.2.3 3D Structural Design Modulation

With optimal printing conditions, it is possible to print several complex constructions with different designs at high resolution, as shown in Figure 4.

**Figure 4:**
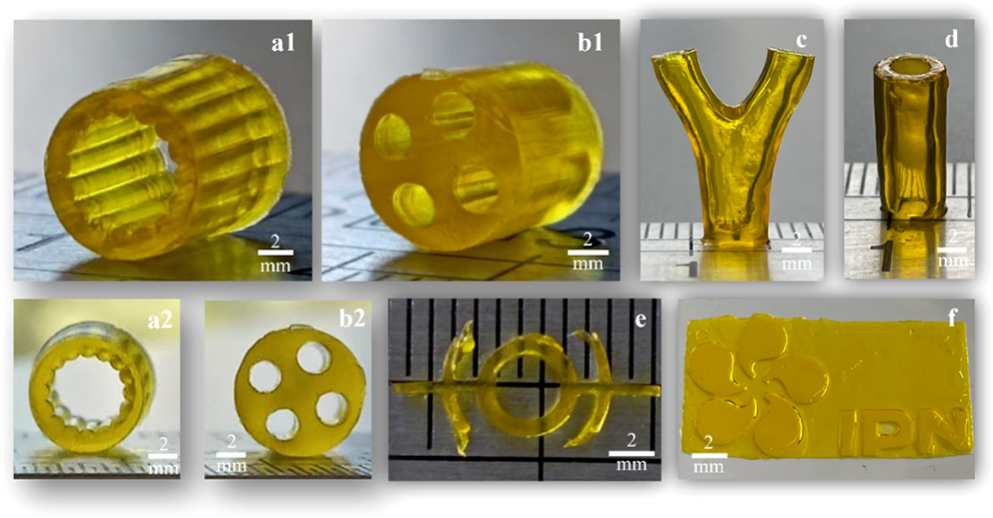
Image of the different complex structures printed by DLP, using Dex/PCL-based biomaterial photoink (20% (w/v) of polymer concentration, 25Dex:75PCL, 0.4% (w/v) LAP, 0.1% (w/v) tartrazine, exposure time 19.25 s, irradiance 21 mW/cm^2^, and layer height of 100 μm). (a1 and a2) Grooved hollow tubes; (b1 and b2) multichannel conduits; (c) branched conduits; (d) hollow conduit; (e) the PolySyc group logo from the Department of Chemical Engineering at the University of Coimbra; and (f) the IPN logo.

Recent studies on DLP photoinks still lack a sufficient variety of structures that they can produce, especially hollow and capillary structures that mimic natural tissue.^40^ In this context, the optimized biomaterial photoink enabled the successful printing of hollow structures (Figure 4c,d), which have the potential for numerous biomedical applications such as artificial blood vessels, nerve guide conduits, and catheters. In addition, it enabled the fabrication of more complex structures, including internal grooves and microchannels (Figure 4a,b) which can promote cell adhesion and axonal growth.^44^ This novel biomaterial photoink expands the possibilities for fabricating complex structures suitable for various biomedical applications.

### 3.3 Characterization of the 3D-Printed Structures

#### 3.3.1 Gel Content

The gel content of each printed tube (*h* × Ø_in_ × Ø_out_: 5 mm × 2.5 mm × 5 mm) was determined. For this purpose, the printed tubes were washed with water, dried under vacuum, and subjected to Soxhlet extraction for 2 days using THF and water as solvents. The gel contents determined for 25Dex:75PCL, 50Dex:50PCL, and 75Dex:25PCL were 92.68 ± 1.04%, 98.71 ± 0.67%, and 98.78 ± 0.31%, respectively. It can be concluded that all the different tubes had high gel content irrespective of the amount of Dex and PCL, indicating an efficient cross-linking process.

#### 3.3.2 Swelling Capacity

The swelling capacity of 3D-printed tubes (*h* × Ø_in_ × Ø_out_: 5 mm × 2.5 mm × 5 mm) was evaluated in PBS (pH 7.4) at 37 °C. Figure 5a illustrates the swelling capacity over time for tubes prepared with different Dex/PCL ratios.

**Figure 5:**
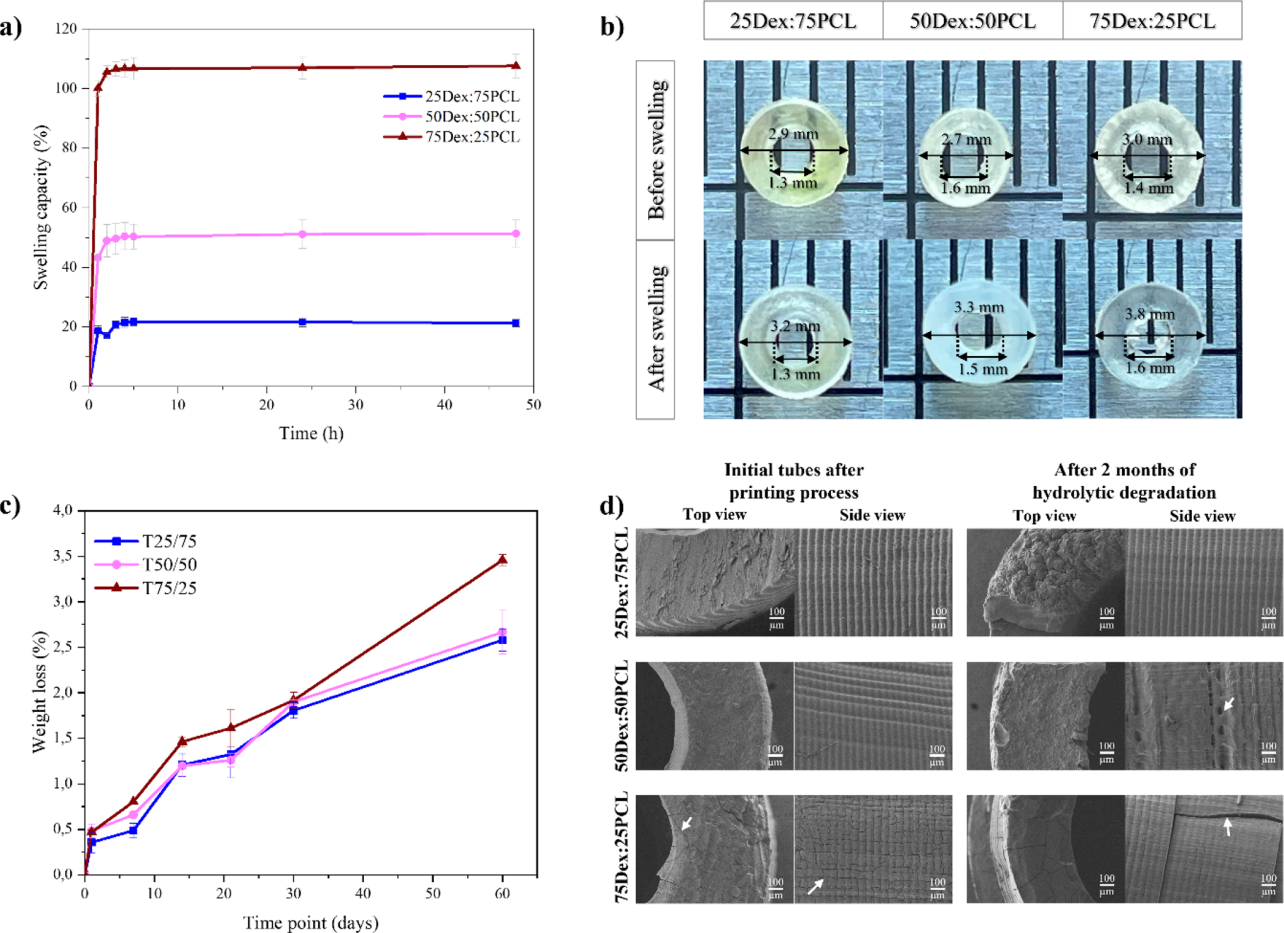
Swelling capacity: (a) Curves of 25Dex:75PCL (blue squares), 50Dex:50PCL (purple dots), and 75Dex:25PCL (red triangles) tubes during 48 h, in PBS (pH 7.4) at 37 °C; (b) dimensions of 3D-printed constructs (*h* × Ø_in_ × Ø_out_: 5 mm × 2.5 mm × 5 mm) after drying at 50 °C (before swelling) and after 48 h immersion in PBS at 37 °C (after swelling). Tube dimensions were measured using ImageJ software. *In vitro* degradation in PBS (pH 7.4) at 37 °C: (c) Curves of the printed tubes for all three formulations: 25Dex:75PCL (blue squares), 50Dex:50PCL (purple dots), and 75Dex:25PCL (red triangles); (d) SEM images of printed tubes with 25Dex:75PCL (first row), 50Dex:50PCL (middle row), and 75Dex:25PCL (final row) formulations after the printing process (left) and after two months of *in vitro* hydrolytic degradation (right). The SEM images represent some cracks on 75Dex:25PCL formulation after the printing process (white arrow), and after hydrolytic degradation, it is possible to observe some pores in the 50Dex:50PCL formulation (white arrow) and some structural defects in the 75Dex:25PCL formulation (white arrow).

The results show that all hydrogels reached their maximum swelling capacity in about 2–3 h. As expected, the 75Dex:25PCL formulation showed the highest swelling capacity (107.65 ± 4.02%), while the other formulations, 50Dex:50PCL and 25Dex:75PCL, showed swelling capacities of 51.39 ± 4.58% and 21.78 ± 1.27%, respectively. This result can be attributed to the high hydrophilicity of dextran and the lower hydrophilicity of PCL.^21,23,45^

It was found that the hydrogel with the highest PCL-IEMA content (25Dex:75PCL) had a lower swelling rate of about 20%. The value of swelling could also influence the shrinkage process after drying the tubes. The percentage of shrinkage in relation to the intended CAD model dimensions must be considered along with the swelling ability of each formulation to adjust the size of the printed constructs. The values for the inner diameter (Ø_in_) and outer diameter (Ø_out_) as well as the design height were measured before and after 48 h of swelling (Figure 5b and Table 4).

**Table 4:** Dimensions of 3D-Printed Constructs before and after Swelling along with the Respective Dimensional and Volumetric Increase^a^,^b^

		Dimensions (mm)			Increment of dimensions (%)			
Formulation	Swelling state	Ø_out_	Ø_in_	*h*	Ø_out_	Ø_in_	*h*	Δ*V*
25Dex:75PCL	Dry	2.79 ± 0.13	1.39 ± 0.01	2.73 ± 0.02	7.89	0.36	8.42	31.80
	Swollen	3.01 ± 0.14	1.38 ± 0.01	2.96 ± 0.03				
50Dex:50PCL	Dry	2.73 ± 0.12	1.37 ± 0.01	2.50 ± 0.03	16.78	8.02	17.61	68.60
	Swollen	3.18 ± 0.08	1.48 ± 0.02	2.94 ± 0.02				
75Dex:25PCL	Dry	3.02 ± 0.02	1.45 ± 0.03	1.62 ± 0.01	23.69	10.07	22.29	98.68
	Swollen	3.74 ± 0.02	1.59 ± 0.01	1.98 ± 0.01				

a Ø_out_: outer diameter of the printed tube, Ø_in_: inner diameter of the printed tube, *h*: height of the printed tube, and Δ*V*: increment of tube volume.

b Measurements were obtained using ImageJ software .

The swelling and shrinkage of the tubes according to the 3 axes correspond to a considerable change in volume. In terms of structural volume, the formulation 25Dex:75PCL swells the least, with its volume increasing by 31.80%, followed by the 50Dex:50PCL formulation, which swells by 68.60%, and finally the 75Dex:25PCL formulation, which swells the most, with a corresponding volume increase of 98.7%, (Table 4). The final size after swelling is an important characteristic, especially for implantable applications. In the context of nerve guide conduits, for example, swelling should be limited to avoid constriction of the nerve.^21^ In our tubes, however, the change in the diameter of the inner hole is minimal, which may be advantageous for implantation applications. The changes in the outer diameter are larger, but they are not as important as those in the inner diameter for this particular application.

#### 3.3.3 *In Vitro* Hydrolytic Degradation

*In vitro* hydrolytic degradation profiles of the tubes (*h* × Ø_in_ × Ø_out_: 5 mm × 2.5 mm × 5 mm) were studied for 2 months at 37 °C using PBS (pH 7.4) containing sodium azide (2% w/v) to inhibit bacterial growth. The behavior of the hydrogels in terms of their hydrolytic degradation is shown in Figure 5c.

Figure 5c shows that after 60 days, none of the printed tubes had degraded by more than 4%. The degradation profiles are similar for the three formulations, indicating that the three formulations have a similar cross-linked structure. Although the weight loss values of all formulations remained close during the first month, a more pronounced degradation was observed for the formulation with a higher dextran content during the second month. The formulation 75Dex:25PCL shows the highest weight loss (3.5%), whereas 50Dex:50PCL and 25Dex:75PCL represent slightly lower values (2.5–3%). This slightly lower degradation in formulations with higher PCL content can be explained by the hydrophobic nature of the precursor PCL-IEMA, which may limit the penetration of PBS into the cross-linked structure.^23^

Although the low level of hydrolytic degradation was observed, SEM images of the tubes after 2 months of degradation were taken and compared with the initial tubes (Figure 5d).

Both 25Dex:75PCL and 50Dex:50PCL showed smooth top and side views with no significant defects. However, in the printed tube with the 75Dex:25PCL formulation, a creature-like texture was observed, defined by cracks (white arrow) measuring 53 μm in height (corresponding to the layer height) and 90 μm in width. The cracks could be related to internal fractures in the printed tube and might lead to poorer mechanical properties. The images of the degradation profile show that the 25Dex:75PCL tube degrades mainly in the cross-section (top view) and has a cauliflower-like surface, while the sidewall of the tube remains relatively intact. The 50Dex:50PCL tube exhibits some surface degradation in the cross-section, but the edge of the wall now appears to be largely affected by concave features between the layer lines and the presence of larger pores (white arrow). As can be seen in Figure 5d, the 75Dex:25PCL again exhibits the crack pattern in both the cross-section and the tube wall, but this time with additional structural defects in the side view (white arrow). As already mentioned, the latter structure degrades about 1% more than the other two formulations after 60 days in the PBS medium.

#### 3.3.4 Thermal Characterization

Table 5 summarizes the temperatures of interest determined from the thermogravimetric curve (TGA), as presented in Figure 6. In relation to the thermal stability of the printed constructs, three stages of weight loss can be identified. The first weight loss, observed below 200 °C, is due to the evaporation of residual moisture and is more pronounced at higher proportions of Dex.^23^ The second stage, occurring between 300 and 350 °C, corresponds to the degradation temperature of the dextran structure,^23^ while the third stage, between 350 and 450 °C, is related to the degradation of the cross-linked PCL-based network.^46,47^ It can also be observed that an increased content of PCL-IEMA in the formulations can lead to an increased thermal stability of the printed constructs.

**Table 5:** Degradation Temperatures Obtained by Thermogravimetric Analysis (TGA) for the Different Printed Cylinders (*h* × Ø_out_: 2 mm × 5 mm) over a Temperature Range of 25–600 °C^a^

Formulation	TG_95%_ (°C)	DTG_peak1_ (°C)	DTG_peak2_ (°C)
25Dex:75PCL	304.9	347.5	419.9
50Dex:50PCL	292.5	332.5	412.5
75Dex:25PCL	162.5	322.4	404.9

a TG_95_: temperature at which 95% of the initial mass remains, DTGpeak1: temperature of the first major degradation, and DTGpeak2: temperature of the second major degradation.

**Figure 6:**
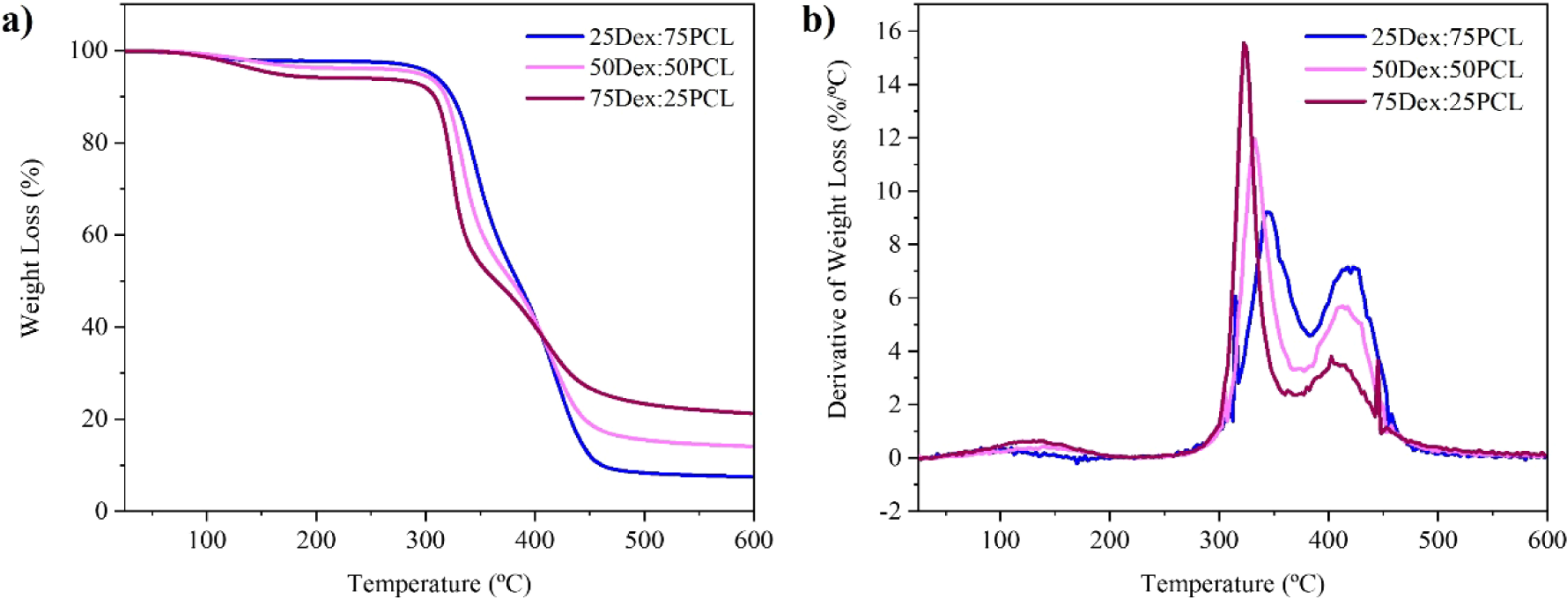
Thermogravimetric (a) curves of printed cylinders (Ø_out_ × *h*: 5 mm × 2 mm) and (b) derivative thermogravimetry for all three formulations (25Dex:75PCL, 50Dex:50PCL, and 75Dex:25PCL) at a temperature range of 25–600 °C.

#### 3.3.5 Contact Angle Measurements

Contact angle measurements were performed for all three printed cylinders (*h* × Ø_out_: 1.5 mm × 20 mm). Although it is a porous material with a swelling capacity between 20 and 100%, measurements were carried out to analyze the surface behavior of the individual formulations. The results showed that the 25Dex:75PCL and 50Dex:50PCL structures exhibited predominantly hydrophobic behavior, with contact angles of 88.74 ± 4.85° and 98.79 ± 5.51°, respectively (no statistical significance). In contrast, the 75Dex:25PCL formulation presented a lower value (70.37 ± 4.07°), indicating a more hydrophilic character, compared to 25Dex:75PCL (** *p* = 0.0084) and 50Dex:50PCL (*** *p* = 0.0009), which is consistent with the higher dextran content. Figure 7 shows the water droplets on the different surfaces.

**Figure 7:**
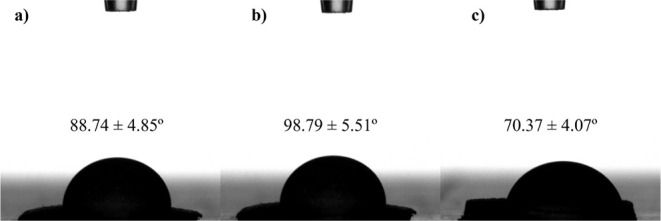
Contact angle measurements on printed cylinders (*h* × Ø_out_: 1.5 mm × 20 mm): Images of a water droplet (3 μL) on the (a) 25Dex:75PCL, (b) 50Dex:50PCL, and (c) 75Dex:25PCL formulations. Distilled water (3 μL) was placed on the printed cylinders (Ø_out_ × *h*: 20 mm × 1.5 mm).

#### 3.3.6 Mechanical Properties

The mechanical properties of the printed cylinders (*h* × Ø_out_: 9 × 6 mm) were also assessed by compression testing. The mean longitudinal compressive stress–strain curves of tubes with different Dex:PCL ratios are presented in Table 6 and Figure 8.

**Table 6:** Compression Results of the Printed Cylinders (*h* × Ø_out_: 9 mm × 6 mm): 25Dex:75PCL, 50Dex:50PCL, and 75Dex:25PCL^a^

Formulation	Compression Stress Max (MPa)	Compression Strain Max (%)	Young Modulus (MPa)
25Dex:75PCL	81.03 ± 2.02	89.27 ± 1.51	16.56 ± 2.70
50Dex:50PCL	68.63 ± 1.00	90.68 ± 4.49	9.10 ± 1.30
75Dex:25PCL	14.22 ± 1.88	86.28 ± 5.46	2.70 ± 0.52

a Measurements were performed on swollen cylinders.

**Figure 8:**
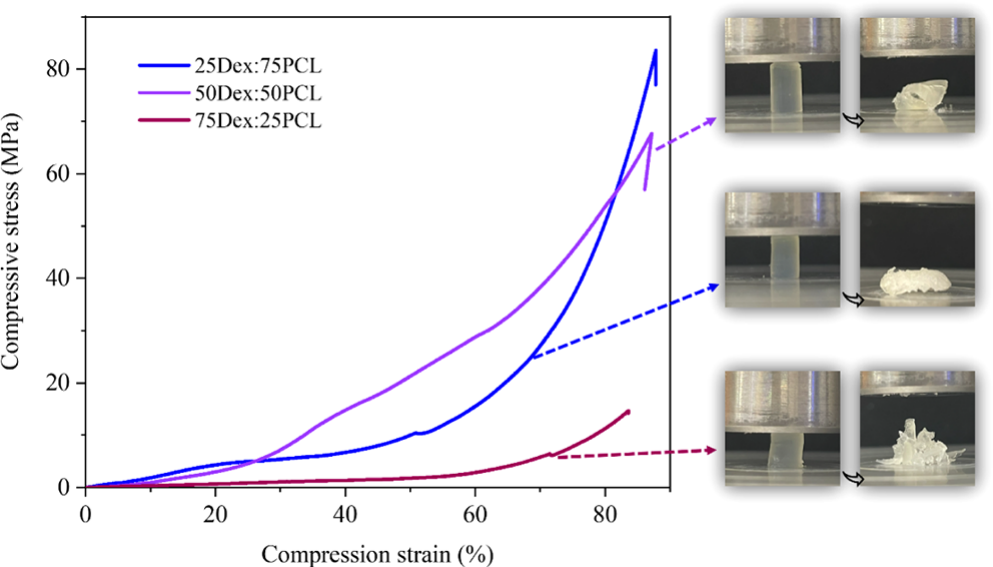
Compressive stress–strain curves of the printed cylinders (*h* × Ø_out_: 9 mm × 6 mm): 25Dex:75PCL, 50Dex:50PCL, and 75Dex:25PCL and respective images of compressed structures.

As expected, the printed structures with less PCL had poor mechanical properties and thus exhibited the lowest values of all formulations for Young’s modulus and compressive stress, while the opposite was true for the construct with a higher PCL content.^48^ However, in terms of compressive strain, all formulations had similar values around 90%, showing that all samples can withstand large deformations before failure, although some are more resistant than others.

For the maximum compressive stress, 25Dex:75PCL has the highest value of 81.03 ± 2.02 MPa, followed by 50Dex:50PCL with 68.63 ± 1.00 MPa, and the lowest value is observed for 75Dex:25PCL with 14.22 ± 1.88 MPa, which shows that the presence of PCL in the formulation determines the structural resistance of the material. There is a significant difference in Young’s modulus between the samples. The 25Dex:75PCL formulation has the highest value of 16.56 ± 2.70 MPa, making it the stiffest and most resistant formulation, followed by the 50Dex:50PCL formulation with 9.10 ± 1.30 MPa, and the weakest formulation, 75Dex:25PCL, with 2.70 ± 0.52 MPa. These results confirm that the incorporation of PCL has successfully enhanced the mechanical properties of our dextran-based material.

Compared to the literature, where most DLP studies focus on GelMA and SF-MA, the reported structures exhibit significantly lower mechanical strength than those presented in this study, with compressive stress values between 0.02 and 1 MPa and compression strain percentages between 40 and 80%, depending on the formulation.^15,17,36^ By incorporating PCL, this study introduces a new generation of biomaterial photoinks that enable the 3D fabrication of structures capable of withstanding higher compressive stresses.

#### 3.3.7 *In Vitro* Cytotoxicity Assay of the Printed Structure Degradation Products

To evaluate the potential cytotoxic effect of the printed cylinders, two different cell lines were incubated at various time points with extracts of the degradation products of the printed structures (indirect method). Figure 9a,b shows the cell viability of HEK293T and Neuro-2a cells, respectively. According to ISO 10993-5:1999, samples with a cell viability of more than 75% can be considered noncytotoxic. As shown in Figure 9a,b, the viability of HEK293T and Neuro-2a cells cultured after different time points was always greater than 75% for all formulations tested, except for 75Dex:25PCL. These results can be explained by the higher amount of methacrylate groups in the formulations with higher Dex-GMA content. Over 14 days, the increased number of methacrylate groups leads to enhanced degradation kinetics^49^ and the formation of −COOH groups through ester hydrolysis, which have been proposed to contribute to some toxicity.

**Figure 9:**
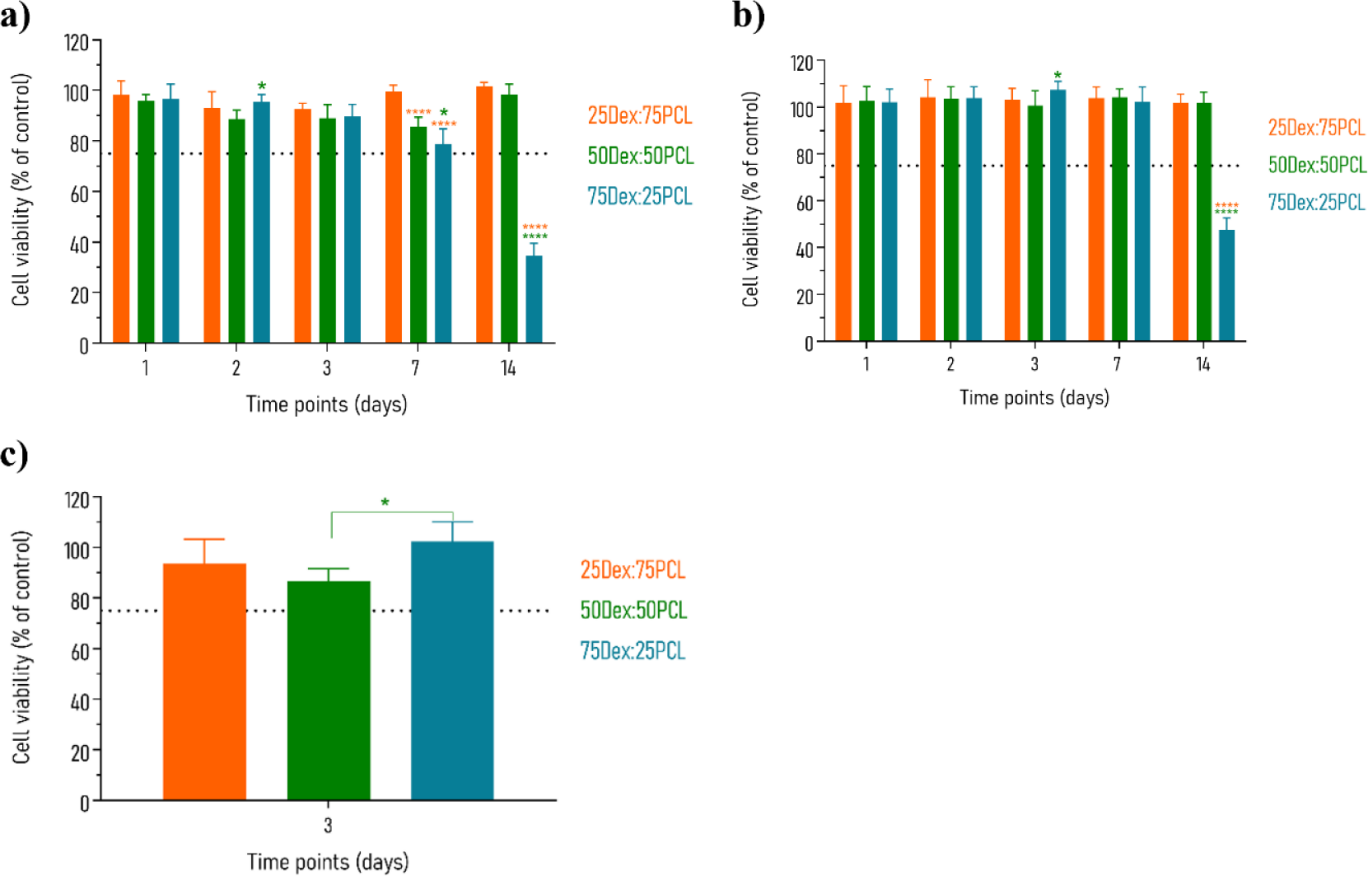
Cell viability results of (**a**) the degradation products of the printed structures in contact with HEK293T cells and (b) Neuro-2a cells. Data represent mean ± SD, *n* = 3. * indicates a significant difference (**** *p* < 0.0001 and * *p* = 0.0350) between samples on the same day of culture and (**c**) the printed structures in direct contact with HEK293T. Data represent mean ± SD, *n* = 3. * indicates a significant difference (* *p* = 0.0105) between the different samples.

The cytocompatibility of the 3D-printed hydrogels was also determined by direct contact. For this purpose, HEK293T cells were seeded on the surface of the hydrogels, and cell viability studies were performed over a 3-day period, as shown in Figure 9c. All 3D-printed hydrogels exhibited high cell viability: 93.72 ± 9.66% of the cells were attached to the 25Dex:75PCL hydrogel, 86.76 ± 4.87% to the 50Dex:50PCL hydrogel, and 102.33 ± 7.84% to the 75Dex:25PCL hydrogel. These data indicate the cytocompatibility of the hydrogels and their ability to support HEK293T cell survival.

## 4 Conclusion

In this work, the development of three different biomaterial photoink formulations based on Dex and PCL for DLP was successfully demonstrated. The combination of these polymers in 3D printing, for the first time in the literature, enabled the fabrication of highly complex structures with tunable mechanical properties, swelling behavior, and degradation profiles.

The study began by optimizing the formulation of the biomaterial photoink, and optimal printability was achieved with 20% polymer concentration, 0.4% LAP, 0.1% tartrazine, and exposure times of 19.25–19.75 s. Despite the excellent print fidelity, shrinkage was observed after processing, especially in samples with a higher PCL content. However, by quantifying the dimensional changes and swelling ratio, the dimensions of the CAD model can be adjusted to achieve the desired final structure for specific applications. The incorporation of PCL also resulted in lower swelling, slower degradation, and enhanced mechanical strength.

In summary, this work has successfully developed a Dex:PCL-based biomaterial photoink that enables the fabrication of highly complex structures, especially hollow architectures, with excellent mechanical strength and controlled biodegradability. These results highlight the potential of the proposed biomaterial photoink for various biomedical applications, including tissue engineering, artificial blood vessels, and nerve guide conduits.

## Supplementary Material



## Notes

^1^H NMR spectra of dextran, dextran-GMA, PCL and PCL-IEMA (Figure S1); equations used to calculate the degree of substitution of Dex-GMA (Equation S1) and PCL-IEMA (Equation S2) (PDF)
