# Supplementary material for: Development of a Tunable Dextran-PCL Biomaterial Photoink for High-Resolution DLP 3D Printing in Biomedical Applications
Source: ACS Appl Mater Interfaces. 2025 Nov 6;17(46):63272–85. doi: 10.1021/acsami.5c18856 (PMC12818721; doi:10.1021/acsami.5c18856)
Supplement: Supplementary file 1 [file am5c18856_si_001.pdf]

SUPPORTING INFORMATION

Development of a Tunable Dextran-PCL Biomaterial Photoink for High-Resolution DLP 3D Printing for Biomedical Applications

Inês C. P. Escobar[a], Leonor Chaves[b], Carlos T. B. Paula[a,b], Patrícia Pereira[a,b], Arménio C. Serra[a], Jorge F. J. Coelho[a,b,]*

[a]University of Coimbra, CEMMPRE, ARISE, Department of Chemical Engineering, Rua Sílvio Lima, Polo II, 3030-790 Coimbra, Portugal

[b]IPN, Instituto Pedro Nunes, Associação para a Inovação e Desenvolvimento em Ciência e Tecnologia, Rua Pedro Nunes, 3030-199 Coimbra, Portugal

***Corresponding author.** Email adress: jcoelho@eq.uc.pt (Jorge Coelho)

S1: Spectra ^{1}H NMR for all compounds and equations for degree of modification

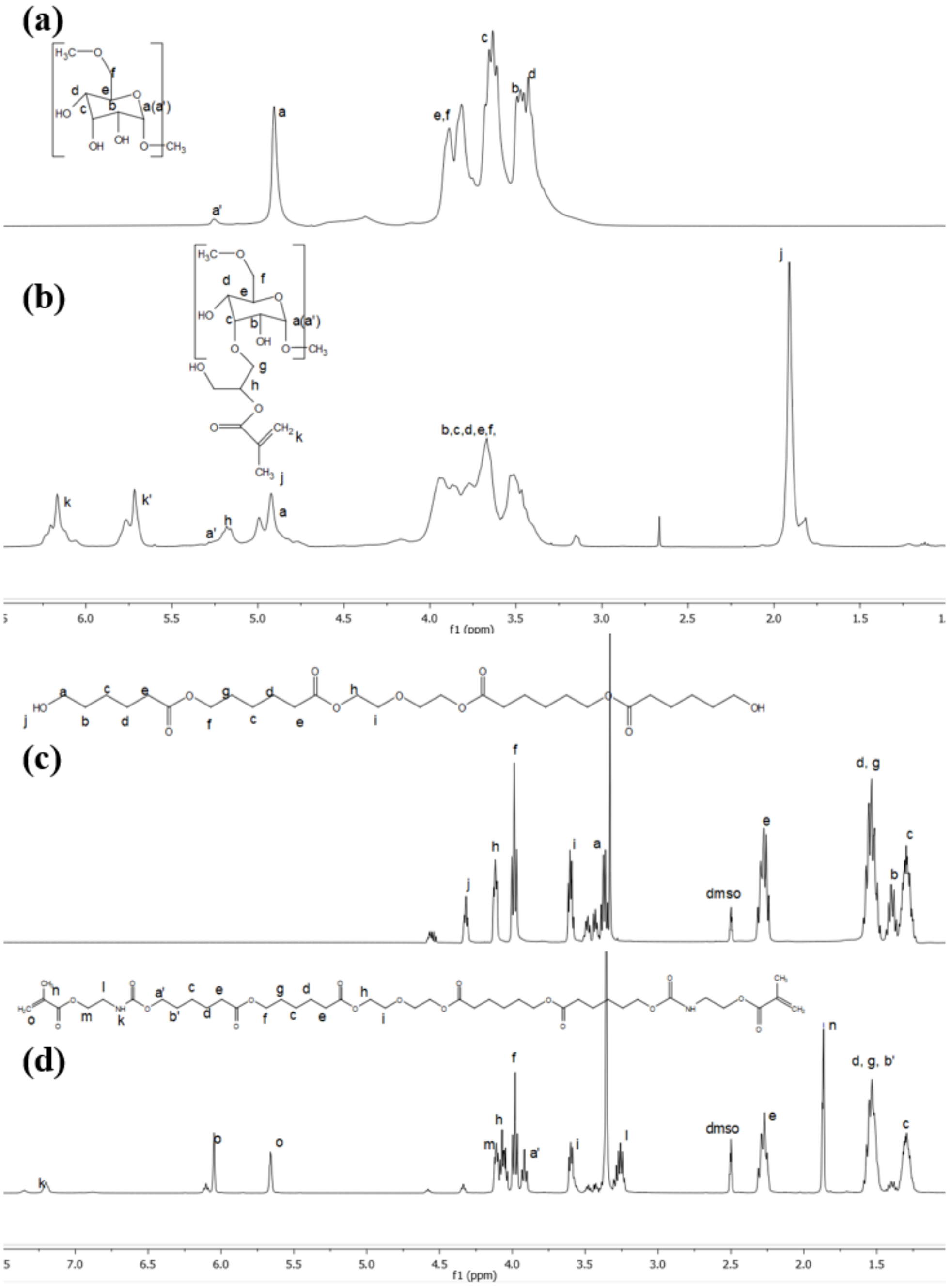

Figure S1. ^{1}H NMR spectra of: (a) PCL and (b) PCL-IEMA; (c) dextran and (d) Dex-GMA at 25 ºC using a Bruker Avance III 400 MHz spectrometer coupled to a 5 mm triple detection TIX probe.

$$DS(Dex - GMA) = \frac{n(GMA)}{n(Dex)} = \frac{\frac{\int k + \int k'}{2}}{\frac{\int a + \int a'}{1}}$$

Equation S1. Equation for calculating the degree of substitution of Dex-GMA, where k and k' correspond to the vinyl protons of GMA (2 H per GMA unit), and a and a' correspond to the anomeric protons of dextran (1 H per dextran repetition unit). DS ranges from 0 to 3.

$$\%PCL - IEMA = \frac{\int \frac{o}{2}}{\int \frac{e}{8}} \times 100$$

Equation S2. Equation for calculating the degree of substitution of PCL-IEMA, where *o* correspond to the vinyl protons of IEMA and *e* to the methylene protons of the PCL repeating unit.
